# Postnatal cord care practices and associated factors in Arba Minch town, southern Ethiopia

**DOI:** 10.1371/journal.pone.0313244

**Published:** 2024-11-14

**Authors:** Zinabu Birhan Teferi, Kasahun Fikadu Tesema, Gebresilasea Gendisha Ukke, Guesh Mebrahtom Tsegay, Freweyni Fisshatsion Yhdego, Goitom Girmay Gebremariam

**Affiliations:** 1 Tigray Health Research Institute, Mekelle, Tigray, Ethiopia; 2 Department of Midwifery, Arba Minch University, Arba Minch, Ethiopia; 3 Health Systems and Equity, Eastern Health Clinical School, Monash University, Box Hill, Victoria, Australia; 4 Department of Adult Health, School of Nursing, Aksum University, Aksum, Ethiopia; 5 Department of Internal Medicine, School of Medicine, Aksum University, Aksum, Ethiopia; 6 Department of Midwifery, Aksum University, Aksum, Ethiopia; Debre Tabor University, ETHIOPIA

## Abstract

**Background:**

Cord care is one component of essential newborn care that reduces newborn morbidity and mortality. Poor cord care practice leads to a large amount of neonatal death due to infection. In Ethiopia, many women give birth at home, where neonates are exposed to unclean cord care practices or application of different traditional substances. Despite the severity of the problem, studies related to postnatal cord care practice are scarce. Hence, this study is aimed to assess umbilical cord care practices and associated factors in the postnatal period in Arba Minch town.

**Objective:**

To assess postnatal cord care practices and associated factors among mothers who gave birth in the last six months in Arba Minch Town, southern Ethiopia, 2019.

**Methods:**

Cross-sectional study design was conducted and using simple random sampling method 423 mothers who gave birth with in the last six months in Arba Minch town from November 11 to December 02, 2019, were selected. Data was collected using structured questionnaire administered by the interviewer. EpiData version 4.4 was used for data entry and SPSS version 25 was used for data analysis. Variables with p ≤ 0.25 in bivarible logistic regressions were considered as potential candidates for multivariable logistic regression analysis to control confounders. Adjusted Odds Ratio with 95% confidence interval was applied in multivariable logistic regression models, to identify variable, which has significant association.

**Results:**

The overall proportion of women who had good postnatal cord care was 67.1%. The maternal age group of 25 to 29 years (AOR = 2.51; 95% CI: 1.35–4.68), maternal educational status of secondary school and above (AOR = 4.19; 95% CI: 2.05–8.54), mothers with good knowledge of cord care (AOR = 1.77; 95% CI: 1.03–3.05), and health facility delivery (AOR = 2.60; 95%CI: 1.05–6.41) were independent factors associated with good cord care.

**Conclusions:**

The proportion of good cord-care practice reported in this study was relatively worthy. Maternal age group of 25–29 years, maternal educational status of secondary school and above, having good knowledge of cord care, and delivering at health facility were factors that increased good postnatal cord care practice. Therefore, investing in improving these factors would positively affect maternal postnatal cord care practice. In order to reduce neonatal morbidity and mortality, routine counseling of cord care practice to mothers would attain good knowledge about postnatal cord care practices. Community health education would also increase awareness and practice of postnatal cord care.

## Introduction

Cord care is one component of essential newborn care that comprises a sequence of steps [1, 2]. Clean and dry cord care is recommended for neonates who are delivered in health institutions and at home. Nevertheless, if the cord stump is exposed to an unclean environment, a high percentage of infections may stem from bacterial colonization of the umbilicus [3]. Application of substances to the cord and/or moistening of the umbilical cord can increase the risk of subsequent cord infection or inflammation of the umbilical cord stump. In developing nations, the risk of umbilical infection can be lowered with the advent of essential interventions such as increasing the provision of tetanus toxoid immunization during pregnancy, providing clean cord care, and avoiding the application of harmful substances to the cord stump [4]. However, in Ethiopia, where most deliveries take place at home, different substances, such as oil, ash, dung, and other unknown ointments, are applied to the newborn umbilical cord stump [5].

Neonatal mortality is a significant public health problem worldwide, accounting for approximately two-thirds of all deaths in infancy and 40% to 47% of under-five deaths [6, 7].

Geographically, neonatal deaths are most prevalent in southern Asia and sub-Saharan Africa, accounting for 39% and 38% of all neonatal deaths, respectively [8]. Approximately 75% of all neonatal deaths occur during the first week of life, and the second most common (28%) cause of death is infection [8]. The infection risk is 62% higher in infants receiving topical cord applications of potentially unclean substances [9].

Omphalitis is an important cause of neonatal mortality, and its prevention is of great importance for public health [10]. The incidence of omphalitis in developed countries is 0.7%, rising to 2.7% in developing countries [10, 11]. The fatality rate of omphalitis ranges between 1% and 15%, depending on the definition used [12]. However, in Nigeria, it was estimated to account for between 10 and 19% of neonatal admissions, and consequently, estimated neonatal deaths were 30–49% [13].

The risk of neonatal mortality and morbidity associated with cord infection can be reduced with the topical application of chlorhexidine. Chlorhexidine is a safe and effective antiseptic used for many years to reduce the risk of acquiring infection in various healthcare settings [14, 15].

In Ethiopia, among the different efforts to reduce neonatal mortality, community-based newborn care was launched in 2012 [16]. It provides a package of community-based interventions with the help of a Health Extension Program (HEP), which includes the promotion of clean and safe delivery, postnatal follow-up of mother and baby, and management of neonatal sepsis by Health Extension Workers (HEWs) when the referral is not possible. Similarly, initiatives to improve newborn care (including safe cord stump care) in health centers and hospitals and the application of chlorhexidine to the cord stump are also ongoing [16]. According to the World Health Organization (WHO) and the Federal Ministry of Health (FMOH) recommendations, the cord stump should be kept dry and clean, and no other substances should be applied. However, in Ethiopia, 15% of neonates are exposed to different harmful substances on their umbilical stump [5]. In the postnatal period, the application of a substance to the cord is the single most important factor driving poor cord care practice [17, 18].

Many deliveries in Ethiopia are practiced at home, where there may be cord care that could be harmful to the neonate. Having an awareness of the current cord care practices has paramount significance. In addition, published works are scarce in this regard. Therefore, this research aimed to assess umbilical cord care given in the postnatal period to neonates in the study area and the corresponding factors associated with postnatal cord care practice.

## Materials and methods

### Study area and period

The study was conducted in Arba Minch town. Arba Minch town is an administrative town of the Gamo zone located in the Southern Nations, Nationalities & People’s Region (SNNPR). It is located in southern Ethiopia, approximately 505 km away from Addis Ababa, the capital city of Ethiopia. The town was administratively divided into four sub-cities and further subdivided into 11 Kebeles (lower administrative level in Ethiopia). The town comprises 23,005 households. The people in the town mainly follow four religious denominations, which are Orthodox, Protestant, Muslim, and Catholic. According to the town’s health office report, the total population of the town was 112,724; of which, 56,587(50.2%) were females. Among the females, 26,265(46.4%) were in the reproductive age group. The mortality rates of children under five, infants, and neonates in the town were 42.8, 33.9, and 18.7 per 1000 live births respectively [19]. In 2019, approximately 1947 newborns were born in half of the fiscal year. The town has one general Hospital and three public health centers including 17 primary and 14 medium private clinics, and 12 drug stores owned by private organizations [20].

The study was conducted from November 11 to December 02, 2019.

### Study design

A cross-sectional study design was applied.

### Population

#### Source population

All mothers who gave birth to a live child in the town of Arba Minch within the six months before and during the study period.

#### Study population

Mothers who gave birth to a live child in the town of Arba Minch within the six months before and during the study period in the selected kebeles.

### Eligibility criteria

#### Inclusion criteria

Mothers who had a baby less than six months old during the study period, and who were residents of Arba Minch town for at least six months before the study period were included.

#### Exclusion criteria

Mothers with known mental health problems and other severe illnesses were excluded.

### Sample size determination

A single population proportion formula was used to estimate the sample size required for the study.


$$n={\left(Z_{/2}\right)}^{2}pq/d^{2})$$

The sample size calculation assumed a 95% confidence level and a 0.05 margin of error. To the best of the authors’ knowledge, there was no similar cross-sectional study available in this area. Hence, a 50% population proportion was used. Adding a 10% non-response rate, the final sample size was estimated to be 423.

### Sampling technique and procedure

**Fig 1:**
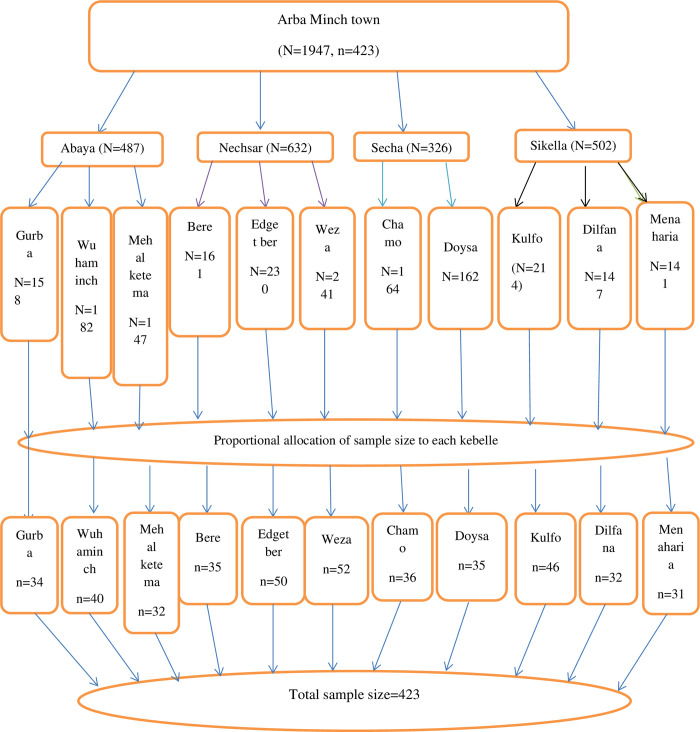
Proportional allocation of samples into kebeles in Arba Minch town, southern Ethiopia 2019.

Arba Minch town has 4 sub cities and 11 kebeles. Namely, Abaya sub city (Gurba kebelle, Wuha Minch kebelle, and Mehal Ketema kebelle), Nech Sar sub city (Bere kebelle, Edget Ber kebelle, and Weza kebelle) Secha sub city (Chamo kebelle and Doysa kebelle) and Sikella sub city (Kulfo kebelle, Dilfana kebelle and Menaharia kebelle)All kebeles were included in the study. First, we communicated with the health extension workers in each kebele. A total of 1947 mothers who gave birth in half of the Ethiopian fiscal year were documented in the town. We obtained a document of all postnatal mothers in each kebele who gave birth in the last six months before the study period. Then, the record was checked for any repeated records, deleted or jumped, and corrected accordingly. Then, we coded each record. Then, as depicted in the figure below, proportional allocation of the sample size (n) based on the population (N) they have was performed, and then frame of women who gave birth in the last six months in each kebelle was prepared. The desired sample size in each kebele was obtained using a simple random sampling technique (Fig 1).

#### Operational definitions

*Knowledge*. Ability to describe the activities of standard cord care practices [21].

*Practice*. This refers to the activities performed and reported by the mother concerning umbilical cord care.

*Good knowledge*. Mothers who scored 50^th^ percentiles were adjusted as having good knowledge.

*Poor knowledge*. Mothers who scored below the 50^th^ percentile have poor knowledge [1].

*Good postnatal cord care practice*. If a mother applies nothing to the cord except Chlorhexidine.

*Poor postnatal cord care practice*. If a mother puts a harmful substance on the cord stump [22].

### Data collection method and procedures

Data were collected using a structured questionnaire adapted from the WHO newborn care component and reviewing other different studies related to postnatal cord care.

Four diploma midwives and one Bachelor of Science (BSc) midwife participated in the data collection process. Prior experience in similar prior work, ability to communicate with the local language, and ability to approach the participants logically were the criteria used to select data collectors and supervisors. The supervisor supervised data collectors closely and, in turn, had regular communication with the principal investigator. After the proportionate sample size for each kebele had been obtained, each participant was identified through a computer-generated simple random sampling method. The data collectors visited the mother in her home with the help of predetermined house numbers and health extension guidance. Overall, 420 mothers participated in the study and gave complete information based on the tool.

### Data quality control

The questionnaire was translated into the local language Amharic by a language expert and then re-translated back to English for consistency. One-day training was given to the data collectors and the supervisor on the data collection tool and data collection procedure by the investigator. During the training, an explanation was given of the objective of the study, and a discussion was made on the tool designed for data collection, and the potential challenges that a data collector could face.

The questionnaire was pretested on five percent (22 mothers) who were living in the ‘Lante’ kebele. The logical accuracy of the instrument, clarity, and simplicity were observed. Both the principal investigator and the supervisor checked the completeness of each questionnaire on a daily basis. The validity of the instrument (questionnaire) was determined through the judgment of subject area experts. Internal consistency was checked by Cronbach’s alpha. The coefficient of Cronbach’s alpha was 0.820. Three professionals who are experts in child health checked it.

### Data processing and statistical analysis

The data were coded, entered using EpiData version 4.4, and exported to SPSS version 25 for statistical analysis. The descriptive values were determined through frequencies and percentages. Then the findings are presented in texts, tables, and graphs.

Multivariable logistic regression analyses were performed at a 95% confidence interval for the existence of an association between the dependent and independent variables. Independent variables with P ≤0.25 in the bivariable model, biological plausibility, and previous research findings were the assumptions used to select candidate variables for the final multivariable logistic regression model. Model fitness was checked using the Hosmer-Lemeshow goodness of fitness test. P-values less than 0.05 with corresponding 95% confidence intervals were used to determine the variables identified as statistically significant associations.

### Ethics approval and consent to participate

The Arba Minch University College of Medicine and Health Sciences Ethical Review Board approved the study (Number: IRB/126/12). The Gamo zonal health office gave us study permission for data collection in Arba Minch town. The aim of the study was explained to the selected study participants. Informed consent was obtained from all study participants. Signed informed consent was obtained from those who could read and write, whereas thumb-printed consent was obtained from those unable to read and write. Verbal informed consent was obtained from the legal guardians/parents. The procedure to obtain thumb-printed consent from participants and verbal informed consent from parents was approved by the Arba Minch University Ethical Review Board. Assent was obtained verbally from study participants under the age of 18 years old. The confidentiality of recruited study participants was maintained throughout the study. All methods were performed following relevant guidelines and regulations.

## Results

### Sociodemographic characteristics

A total of 420 mothers participated in the study. This gives a response rate of 99.3%. The mean age of the study participants were 25.5 years with standard deviation of ±4.388. The majority of the participants were in the age group of 25–29 years. One-third of participants (35.2%) had a primary educational level (Table 1).

**Table 1:** Distribution of study subjects based on sociodemographic variables in the last six months in Arba Minch town, Ethiopia 2019 (n = 420).

Variables	Response	Frequency	Percent (%)
**Age**	15–19	29	6.9
	20–24	147	35
	25–29	162	38.6
	30–34	64	15.2
	≥35	18	4.3
**Religion**	Orthodox	232	55.2
	Protestant	148	35.2
	Muslim	30	7.1
	Others**	10	2.3
**Ethnicity**	Gamo	280	66.7
	Amhara	67	16
	Gofa	44	10.5
	Wolaita	12	2.9
	Other*	17	4
**Marital status**	Married	397	94.5
	Divorced	12	2.9
	Widowed	6	1.4
	Single	5	1.2
**Educational status**	Primary education	148	35.2
	Secondary education	114	27.1
	College & above	81	19.3
	Unable to read and write	77	18.3
**Maternal occupation**	Housewife	220	52.4
	Merchant	117	27.9
	Government employee	48	11.4
	Private employee	35	8.3
**Family monthly income**	<1500ETB	209	49.8
	≥1500ETB	211	50.2
**Distance to the nearest facility**	<30 minutes	355	84.5
	≥30 minutes	65	15.5

* Konso = 7(1.7%), Oromo = 6(1.4%), Zeiss = 3(0.7%), Gurage = 1(0.2%)

** Adventist = 6(1.4%), Catholic = 4(0.95%).

### Knowledge of mothers on cord care

This study revealed that the majority (79.3%) of the study participants had an awareness of cord care practice. It also showed that half (50.7%) of the participants were found to have good knowledge of cord care practices. Regarding the source of information, 65.2% of the study participants sources of information were non-health workers.

### Maternal health service and obstetric characteristics

Of all the study participants, 89% were delivered in health facilities. Almost all respondents (99%) had antenatal care (ANC) visits. However, only 10.6% of study participants were counseled about cord care practice during their visits (Table 2).

**Table 2:** Maternal health service and obstetric characteristics of respondents in the last six months in Arba Minch town, southern Ethiopia, 2019 (n = 420).

Variable	Responses	Frequency	Percent (%)
**Delivery place (n = 420)**	Health facility	374	89
	Home	46	11
**Number of ANC visits (n = 416)**	One visit	11	2.6
	Two visits	27	6.5
	Three visits	62	14.9
	Four visits	316	76
**Counseled on PNCCP during ANC(n = 416)**	Yes	44	10.6
	No	372	89.4
**Number of PNC visits**	0–24 hours	377	89.8
	2^nd^ -3^rd^ days	129	30.7
	7^th^ -14^th^ days	22	5.2
	At 6^th^ week	312	74.3
**Parity of respondents(n = 420)**	Primipara	152	36.2
	Multipara	241	57.4
	Grand multipara	27	6.4

PNCCP = postnatal cord care practice

### Postnatal cord care practice

A majority of the study participants (67.1%) had good postnatal cord care practices. However, the remaining study participants (32.9%) applied traditional substances to the cord stump. The main substance applied was butter. Forty-seven percent of women had referred their mothers to apply the substance on their child’s cord. Most (92%) of the substance was applied within the first week of age of the newborn (Table 3).

**Table 3:** Characteristics of women who applied substances on *the* cord in the last 6 months in Arba Minch town, southern Ethiopia, 2019.

Variable	Responses	Number	Percent (%)
**Substance Applied**	Yes	138	32.9
	No	282	67.1
**Person applying the substance(n = 138)**	Myself	116	84.1
	my mother	18	13
	my grandmother	3	2.2
	Mother-in-law	1	0.7
**Reference to apply the substance(n = 138)**	my mother	65	47.1
	My experience	22	16.0
	Neighbor	19	13.8
	Grandmother	17	12.3
	Mother-in-law	15	10.8
**Age of the baby during the application (n = 138)**	0–7 days	127	92.0
	8-15days	11	8.0
**Frequency of application (n = 138)**	Once	81	58.7
	Twice	45	32.6
	Three times	12	8.7

### Characteristic of substances applied to the umbilical cord

**Fig 2:**
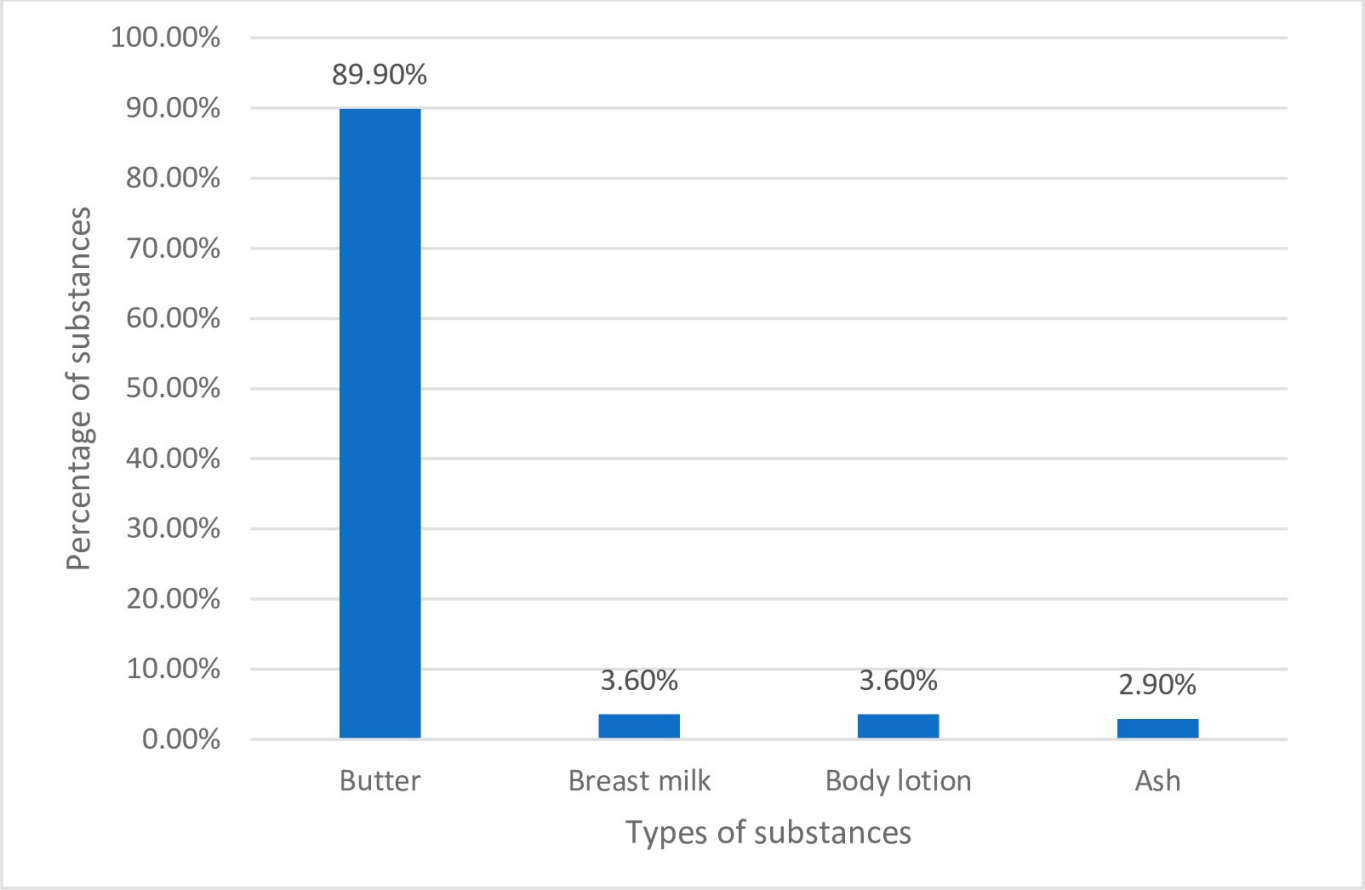
Distribution and types of substance applied by mothers on their babies’ cords in the last six months in Arba Minch town, Ethiopia 2019.

Butter was the frequently mentioned substance to be applied to the umbilical cord in the current study (Fig 2).

**Fig 3:**
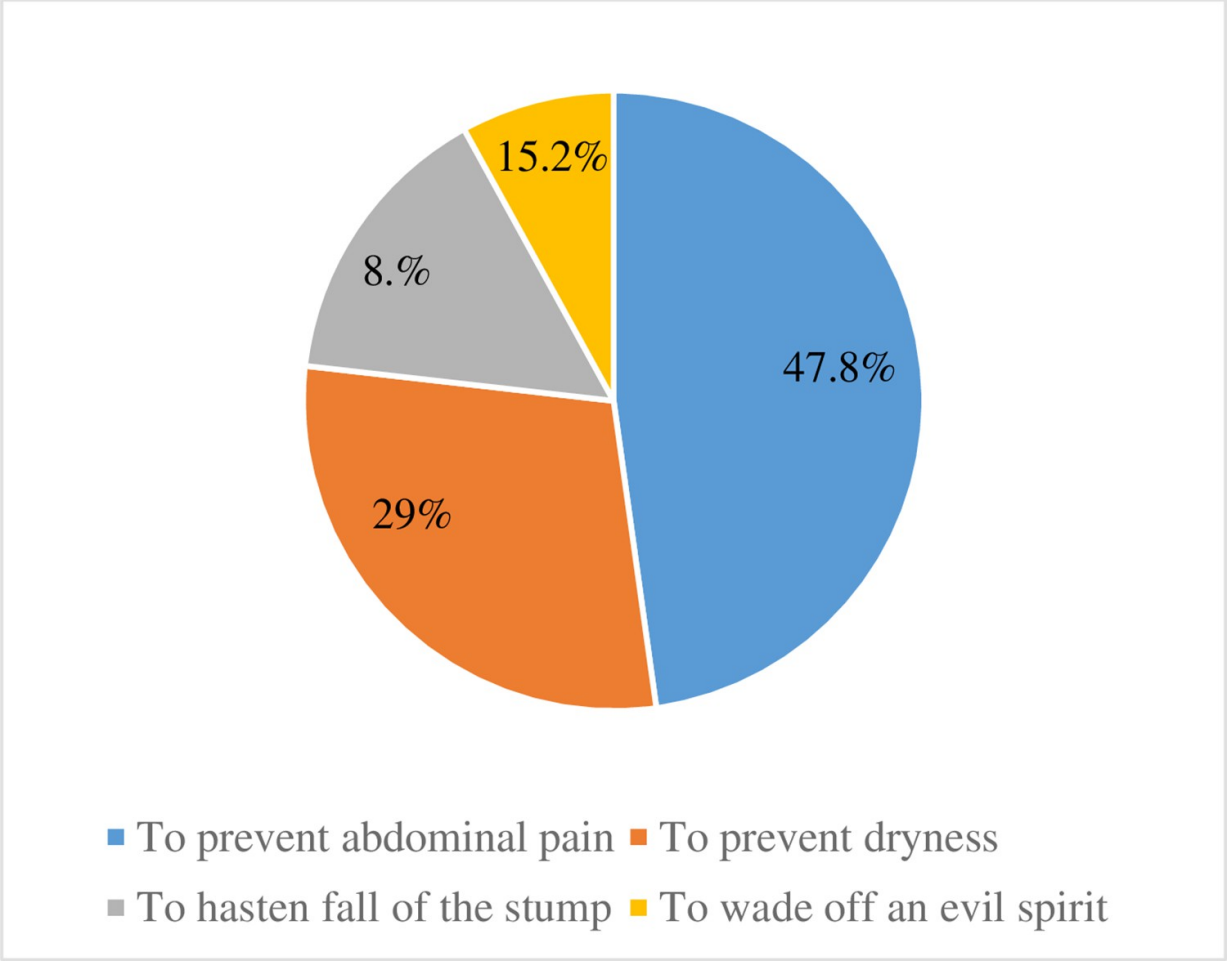
Reason for respondents to apply substances on the cord stump in the last six months in Arba Minch town, Southern Ethiopia 2019.

The commonly mentioned reason why mothers had applied substances on their babies’ umbilical cord was to prevent abdominal pain followed by mothers who believed that substance application could prevent umbilical dryness, which accounts for approximately, 48% and 29% respectively (Fig 3).

Most of the mothers 368(87.6%) had washed their hands before giving care of their baby’s umbilical cord stump. Almost all of the mothers 410(97.6%) hugged their baby after dressing it in cloth, and only two mothers reported skin-to-skin contact, while the remaining eight mothers reported separated beds for the newborn. The majority (75.5%) of the respondents also reported that they bathed their baby before the stump cut off by immersing it in prepared water, and only one (0.2%) mother reported that she used a sponge bath. In comparison, the remaining 102(24.3%) used pouring water on it. Regarding the dressing of their baby’s cord stump, either with a light clean cloth or leaves uncovered was reported in 323(76.9%) mothers. The average time of cord stump separation was 7.33 days, ranging from four to seventeen days. The proportion of mothers whose baby’s umbilical cord stump faced a problem was 14(3.3%). The types of problems that were mentioned were pussy discharge, wounding, bleeding, and redness around the stump at 1.2%, 1%, 0.7%, and 0.5%, respectively. Eight (57.1%) of the babies with cord problems were managed at home while the remaining others were taken to a health facility.

### Factors associated with cord care practice

Variables included in the multivariable logistic regression analysis were those variables with P ≤0.25 at bivariable logistic regression analysis. The variables with P ≤0.25 at bivariable logistic regression analysis were age of the mother, maternal educational status, knowledge level of the mothers, awareness, husband education, place of delivery, postnatal care visit and distance to the health facility. On multivariable binary logistic regression analysis, the age of the mother, place of delivery, knowledge level of mothers, and maternal educational status were the independent factors significantly associated with postnatal cord care practice. Mothers in the age group of 25–29 were found to be 2.5 more likely to give good postnatal cord care than those 30 and above years old (AOR = 2.5; 95% CI: 1.35–4.70). This study showed that mothers who gave birth at a health facility were 2.6 times more likely to practice good postnatal cord care than those who gave birth at home (AOR = 2.6; 95% CI:1.05–6.41).

Similarly, the odds of engaging in good cord care practice were 4.2 among mothers who had completed secondary school and above educational level than those who had no formal education (AOR = 4.2 at 95%CI: 2.05–8.54).

Mothers with good knowledge have good practices approximately 2 times more often than those with poor knowledge about cord care (AOR = 1.77; 95% CI: 1.03, 3.05) (Table 4).

**Table 4:** Factors associated with postnatal cord care practice in Arba Minch town, southern Ethiopia, 2019 (n = 420).

Variable	Postnatal cord care practice		COR(95%CI)	AOR(95%CI)
	Good	Poor		
Maternal age				
<20	20	9	2.12(0.86–5.20)	1.96(0.66–5.81)
20–24	97	50	1.85(1.07–3.21)	1.44(0.73–2.83)
25–29	123	39	3.00(1.71–5.28)	2.51(1.35–4.68)**
> = 30	42	40	1	1
Maternal education				
No formal education	36	41	1	1
Primary school	93	55	1.93(1.10–3.37)	1.83(0.95–3.52)
Secondary & above	153	42	4.15(2.36–7.29)	4.19(2.05–8.54)***
Maternal occupation				
Housewife	140	80	1	1
Governmental employee	33	15	1.26(0.64–2.45)	0.83(0.37–1.85)
Merchant	87	30	1.66(1.01–2.73)	1.37(0.75–2.48)
Private employee	22	13	0.97(0.46–2.02)	0.69(0.28–1.70)
Source of information				
Health workers	108	38	1.63(1.05, 2.55)	1.18(0.60–1.93)
None health workers	174	100	1	1
Counseled on cord-care				
Yes	39	12	1.69(0.85–3.33)	1.21(0.51–2.88)
No	243	126	1	1
Knowledge on cord-care				
Good	160	53	2.10(1.39–3.19)	1.77(1.03–3.05)*
Poor	122	85	1	1
Awareness				
Yes	234	99	1.92(1.18–3.11)	0.92(.48–1.78)
No	48	39	1	1
Husband education				
Have formal education	260	116	2.20(1.19–4.21)	1.32(0.62–2.78)
no formal education	22	22	1	1
Place of delivery				
Health facility	265	109	4.20(2.19–7.86)	2.60(1.05–6.41)*
Home	17	29	1	1
Postnatal care visit				
Yes	275	126	3.74(1.44–9.73)	0.93(0.25–3.48)
No	7	12	1	1
Distance to the health facility				
<30 minutes	243	112	1.45(0.84–2.49)	0.99(0.53–1.85)
≥30 minutes	39	26	1	1

Key

***significance at p-value <0.001

**significant at p-value <0.01

*significant at p-value <0.05.

## Discussion

In this study, the majority of the practices were found to be in line with the recommendations of the WHO, which is no application of harmful substances [23]. The study shows that the proportion of mothers who practiced good postnatal cord care practice was 67.1%, which is lower than the study finding in Nigeria (77.8%) [1]. Socioeconomic factors and traditional and cultural variations may contribute to the differences.

In contrast, this finding was higher than other studies conducted in India 44.2% [24], urban slum India 7% [25], Rohtak Haryana 60% [26], Nigeria 58.3% [27], Parakou Benin 9.5% [28], Tabora region 21% [29], Nigeria 54.4% [30], Ghana 35.7% [31] and Uganda 51% [17]. This difference could be attributed to most of the study participants of this study giving birth at health facilities, in which the application of substances is less likely [1, 26, 32]. Another possible explanation might be the socio-demographic, cultural, and traditional divergences, and this study was conducted in an urban community where better awareness is more likely.

In this study, the age of the mother, delivery in the health facility, knowledge level of mothers, and maternal educational status were found to have a significant association with postnatal cord care practice. Mothers in the age group of 25–29 were found to be 2.5 more likely to practice good postnatal cord care than those 30 and above years old. This finding contradicted the study findings in Benin City, Nigeria, which revealed that older mothers practiced good cord care more [33]. The difference could be due to the varying educational levels of the respondents, ease of access to health information, place of delivery, and cultural affiliations, among others.

This study revealed that mothers with good knowledge about cord care were more likely to practice good postnatal cord care than those with poor knowledge. This is in line with a study performed in Nepal [34] as well as a study conducted in Nigeria [1]. Good cord care practice among the respondents with good knowledge about cord care was higher than among those with poor knowledge. The reason could be that mothers with good knowledge may have more health service utilization behavior and could differentiate the harmful and beneficial activities provided for their babies.

This study showed a significant association between mothers who had an educational level above secondary school and postnatal cord care practice. This is consistent with the study conducted in Bayelsa State Nigeria, Edo State Nigeria, and Rohtak Haryana [1, 26, 32]. The provision of appropriate postnatal cord care increases with increasing educational levels. This might be because educated mothers increase the tendency to obtain services and to read materials related to baby care. However, this was not in line with a study conducted in Nepal where a significant association was not found between educational status and the application of substance [35]. This is probably attributed to the place of delivery, residence, cultural and traditional conditions, and socioeconomic differences.

In this study, a significant association was also observed between the place of delivery and postnatal cord care practice. Mothers who gave birth to their current baby at health facilities were more likely to practice good postnatal cord care than those who gave birth at home. This was consistent with different studies conducted in Nigeria [1, 32]. The reason is probably due to the exposure to health service utilization of the mothers could develop their awareness of newborn care practice.

## Conclusions

The proportion of mothers who had given good postnatal cord care practice was found to be 67.1%. Even though the proportion of good cord care practice is comparatively good, there is still a significant problem with using potentially harmful substances for cord care.

Maternal age, level of education, knowledge of cord care practice, and health facility delivery were found to have a significant statistical association with postnatal cord care practice. Routine cord care counselling throughout antenatal care, delivery, and postnatal care visits to mothers would provide them with good understanding of postnatal cord care practices, enabling them to practice successfully and reduce infant morbidity and death. Community health education would also increase awareness and practice of postnatal cord care.

### Strengths and limitations of the study

#### Strengths

The sample is more representative as it was drawn from the community.

The fact that we used primary data will improve the data’s quality.

#### Limitation

Since the data was gathered from mothers who gave birth six months ago, recall bias may have existed.
